# A Study on the Timing Sensitivity of the Transient Dose Rate Effect on Complementary Metal-Oxide-Semiconductor Image Sensor Readout Circuits

**DOI:** 10.3390/s24237659

**Published:** 2024-11-29

**Authors:** Yanjun Fu, Zhigang Peng, Zhiyong Dong, Pei Li, Yuan Wei, Dongya Zhang, Yinghong Zuo, Jinhui Zhu, Shengli Niu

**Affiliations:** 1 State Key Laboratory of Intense Pulsed Radiation Simulation and Effect, Northwest Institution of Nuclear Technology, Xi’an 710024, China; fuyanjun@nint.ac.cn (Y.F.); zhangdongya@nint.ac.cn (D.Z.); zuoyinghong@nint.ac.cn (Y.Z.); zhujinhui@nint.ac.cn (J.Z.); niushengli21@sohu.com (S.N.); 2 Department of Nuclear Science and Technology, Xi’an Jiaotong University, Xi’an 710049, China; pengzg@stu.xjtu.edu.cn (Z.P.); dongzhiyong@stu.xjtu.edu.cn (Z.D.)

**Keywords:** CMOS image sensor, readout circuit, transient dose rate effect, timing sensitivity, fault injection

## Abstract

Complementary Metal-Oxide-Semiconductor (CMOS) image sensors (CISs), known for their high integration, low cost, and superior performance, have found widespread applications in satellite and space exploration. However, the readout circuits of pixel arrays are vulnerable to functional failures in complex or intense radiation environments, particularly due to transient γ radiation. Using Technology Computer-Aided Design (TCAD) device simulations and Simulation Program with Integrated Circuit Emphasis (SPICE) circuit simulations, combined with a double-exponential current source fault injection method, this study investigates the transient dose rate effect (TDRE) on a typical readout circuit of CISs. It presents the variations in the photoelectric signal under different dose rates and at different occurrence moments of the TDRE. The results show that, under low dose rates, the CIS readout circuit can still perform data acquisition and digital processing, with the photoelectric signal exhibiting some sensitivity to the occurrence moment. At high dose rates, however, the photoelectric signal not only remains sensitive to the occurrence moment but also shows significant discreteness. Further analysis of the CIS readout circuit sequence suggests that the occurrence moment is a critical factor affecting the circuit’s performance and should not be overlooked. These findings provide valuable insights and references for further research on the TDRE in circuits.

## 1. Introduction

Due to the high integration density, low cost-effectiveness, and outstanding performance, complementary Metal-Oxide-Semiconductor image sensors (CISs) has been extensively developed in many applications, such as satellite remote sensing and space exploration [1,2,3]. However, when operating in complex space environments or highly radioactive surroundings, CISs are vulnerable to ionizing radiation effects, particularly transient dose rate effects (TDREs), which can cause functional malfunctions or even lead to system-wide failures. These challenges significantly impact the stability and reliability of satellite systems [4,5].

Therefore, research on the key circuits of CISs is essential for providing foundational data to assess their overall damage response to radiation. The readout circuit, a core component of CISs, is responsible for converting photosensitive electrical signals from pixel units into binary digital signals [6,7,8]. Any failure in the readout circuit under radiation conditions may lead to a decline in image quality, manifesting as blurring, distortion, or even complete image capture failure [9,10]. Current research on the radiation effects in CISs has primarily focused on 4T pixel units. For example, Wang, Z. et al. conducted a series of experiments to investigate the degradation mechanisms of 4T image sensors under ^60^Co γ and proton irradiation [11,12,13,14]. O. Marcelot et al. used Technology Computer-Aided Design (TCAD) simulations to examine the dark current sharing and cancellation mechanisms in 4T pixel units [15,16,17]. Additionally, many studies have concentrated on optimizing the circuit structure of CISs to meet the demands for high-speed data acquisition or high-quality image capture [18,19,20]. However, there has been relatively little research on the effects of the TDRE in CISs, particularly in relation to the dynamic process of these effects. The TDRE refers to the radiation effect of high-dose-rate transient-pulse-radiation-irradiating semiconductor devices in a short period of time, generating excess charge carriers. The excess charge carriers are collected at the PN junction through drift and diffusion processes, forming transient photocurrents, which ultimately affect the performance of the device.

In this paper, the TDRE of a typical CIS readout circuit is studied using TCAD device simulations and Simulation Program with Integrated Circuit Emphasis (SPICE) circuit simulations, along with a double-exponential current source fault injection method. Based on the working timing sequence of the CIS, the influence of different dose rates and the occurrence moment of the TDRE on the photoelectric signal is analyzed. The dose rate range of γ radiation refers to the commonly used dose rate range in transient γ irradiation experiments [20], 1 × 10^6^–1 × 10^11^ rad(Si)/s. Additionally, the dynamic response of the photoelectric signal is preliminarily discussed.

## 2. Model and Methodology

### 2.1. Constructing a Typical Model of the CIS Readout Circuit

**Figure 1:**
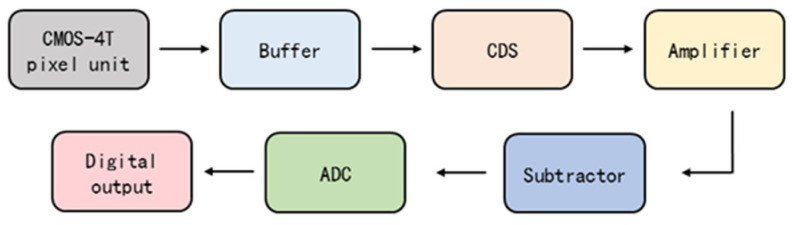
Schematic diagram of the typical CMOS pixel unit readout circuit structure.

As depicted in Figure 1, a netlist model of a typical CIS readout circuit was constructed using circuit simulation software SPICE L-2016.03-2. The model, based on a 90 nm Metal-Oxide-Semiconductor Field-Effect Transistor (MOSFET) process, includes a 4T pixel unit, a correlated double sampling (CDS) circuit, a subtractor circuit, and a digital-to-analog converter (ADC). The operation sequence of the CIS readout circuit is as follows: First, the 4T pixel unit is illuminated, generating a photoelectric signal, V_4T_OUT_, which includes the reset signal (V_CDS_RST_) and the sampling signal (_VCDS_CT_). Next, the V_4T_OUT_ signal is routed to the CDS circuit to mitigate low-frequency noise [21]. The signal is then transferred to the subtractor circuit, where the effective photoelectric signal, V_CDS_R-C_, is derived by calculating the variation between V_CDS_RST_ and V_CDS_CT_. Finally, the V_CDS_R−C_ signal is converted into a machine-readable binary digital signal, V_4T_OUT_ADC_, by the ADC circuit. To minimize interference between adjacent circuit modules, a buffer and amplifier circuit is deliberately incorporated [22]. Due to the scope of this paper, the specific structure of the readout circuit is not presented; however, details on the principle and structure can be found in the referenced literature [6,23,24].

**Figure 2:**
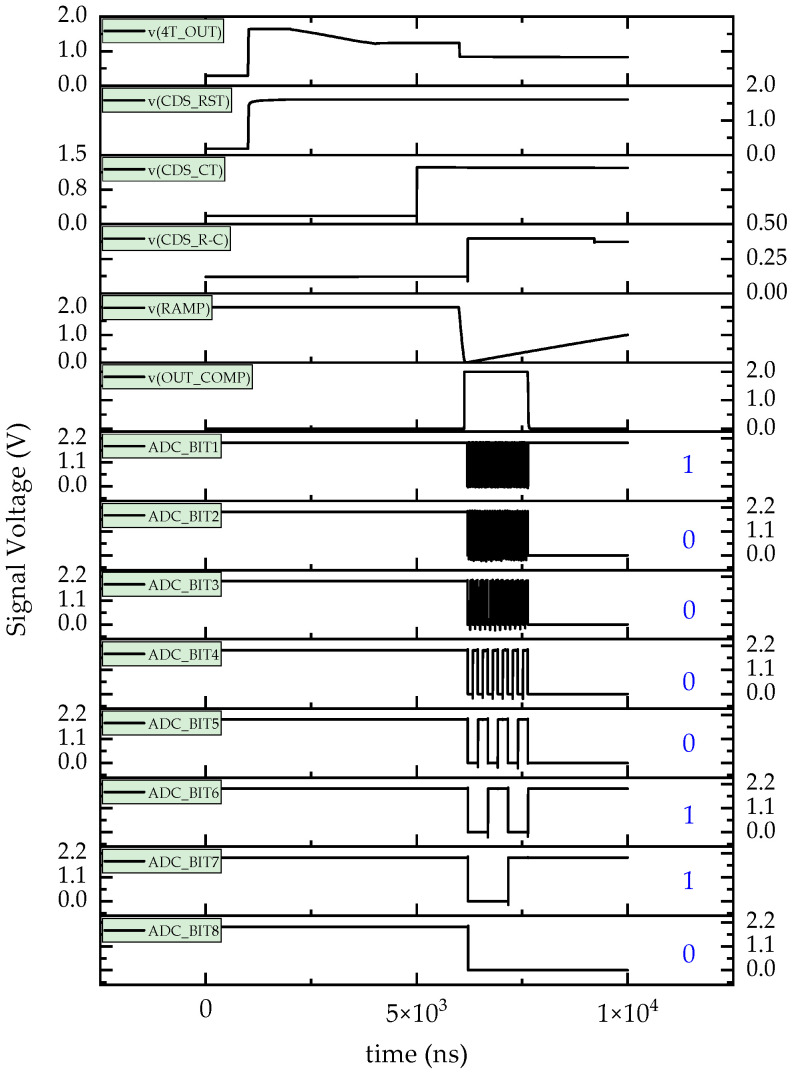
Simulation results of the typical CMOS pixel unit readout circuit output under non-irradiated conditions.

Based on the constructed netlist simulation model of the typical CIS readout circuit, the normal (non-irradiated) state of the circuit was tested to further validate its functionality. The operation timing was set to 10 µs. As shown in Figure 2, the values of V_CDS_RST_ and V_CDS_CT_ were 1.605 V and 1.223 V, respectively, when the V_4T_OUT_ signal was processed by the CDS circuit. In the subtractor circuit, the V_CDS_R−C_ was calculated to be 0.39 V. Subsequently, the V_CDS_R−C_ signal was compared with the ramp voltage, VRAMP, from the ADC circuit (with a ramp amplitude of 1.0 V). As V_RAMP_ increased in parallel with V_CDS_R−C_, the comparator output, V_OUT_COMP_, switched from a high to a low state. The value of V_CDS_R−C_ was thus represented by the pulse width of the V_OUT_COMP_ signal. Finally, the V_OUT_COMP_ signal was converted into an 8-bit digital signal, V_4T_OUT_ADC_, with the value “01100001”.

The digital V_4T_OUT_ADC_ signal accurately represented the initial V_CDS_R−C_ (~0.39 V) and was encoded as “01100001”, which corresponded to a value of 0.38 V when decoded to V_4T_OUT_ADC_10_ (decimal). The calculation is as follows: 0 × 2^7^ + 1 × 2^6^ + 1 × 2^5^ + 0 × 2^4^ + 0 × 2^3^ + 0 × 2^2^ + 0 × 2^1^ + 1 × 2^0^ = 97, and the voltage was derived by 1.0 V × (97/(2^8^ − 1)) = 0.38 V, where the number with an underscore indicates the signal of V_4T_OUT_ADC_, and 1.0 V represents the ramp amplitude. The close match between the V_CDS_R−C_ and V_4T_OUT_ADC_10_ values confirms the accuracy of the typical CIS readout circuit described, supporting its potential use as a foundational simulation model for studying the TDRE.

### 2.2. Simulation Methodology of the TDRE

**Figure 3:**
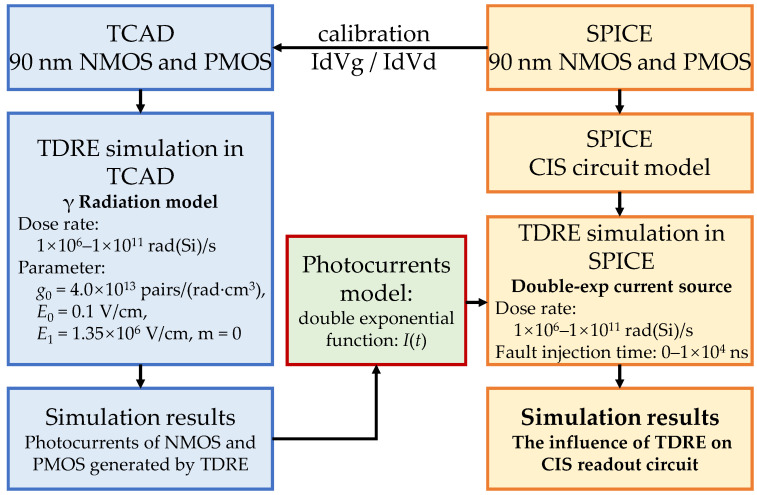
Simulation process of the TDRE in CMOS image sensors.

The TDRE simulation of CMOS image sensors includes TCAD device simulation and SPICE circuit simulation, as shown in Figure 3. Detailed introductions of the two simulation methods are provided in Section 2.2.1 and Section 2.2.2, respectively.

#### 2.2.1. Calculation of the Photocurrent Induced by the TDRE

**Figure 4:**
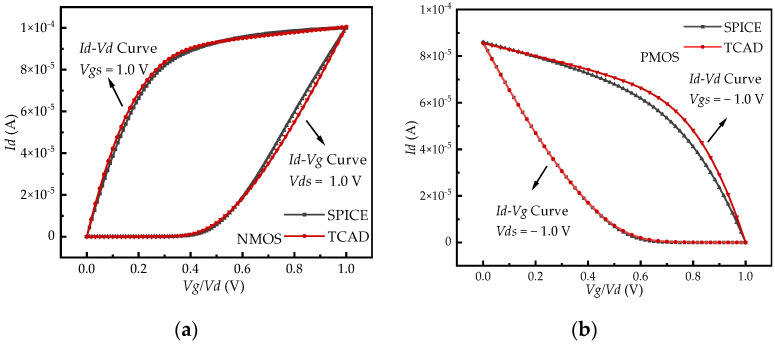
Comparison of the (**a**) NMOS and (**b**) PMOS IV characteristic curves between TCAD simulation results and SPICE analytical results.

The accuracy of the geometric model for MOS devices is crucial for obtaining the photocurrent induced by the TDRE. The specialized TCAD Sentaurus software O-2018.06-SP2 [11], developed by Synopsys, is one of the most powerful tools for simulating the technological and electrophysical characteristics of semiconductor structures and devices. With its built-in radiation models, Sentaurus TCAD has become a leading tool for simulating radiation effects. In this study, a three-dimensional model of both N-Channel MOSFET (NMOS) and P-Channel MOSFET (PMOS) devices was constructed using the 90 nm process technology in Sentaurus TCAD [25]. As shown in Figure 4, by adjusting key parameters such as geometric dimensions and doping concentrations, the simulation results—represented by the *Id*-*Vg* and *Id*-*Vd* characteristic curves—closely matched the SPICE analytical compact model, thereby verifying the accuracy of the MOS device model.

In this paper, the primary photocurrents were calculated using the gamma radiation physical model in TCAD, which is widely used to simulate the TDRE [26,27]. In this model, the electron–hole (e–h) pair generation rate (*G_r_*) is derived as a linear function of the dose rate (*D*), and it depends on both the e-h creation rate (*g*_0_) and the yield function (*Y*(*F*)). The generation rate (*G_r_*) is calculated using the following Equation (1):

(1) $$G_{r}=g_{0}\times D\times Y\left(F\right)$$

(2) $$Y\left(F\right)={\left(\frac{F+E_{0}}{F+E_{1}}\right)}^{m}$$

A wide range of dose rates, from 1 × 10^6^ to 1 × 10^11^ rad(Si)/s, was used for TDRE simulations in Sentaurus TCAD, with a fixed temperature of approximately 300 K and pulse duration of about 10 ns. For Si-based semiconductors, the following constants were used: *g*_0_ = 4.0 × 10^13^ pairs/(rad·cm^3^), *E*_0_ = 0.1 V/cm, *E*_1_ = 1.35 × 10^6^ V/cm, m = 0. Consistent with the references [27], for the NMOS device, the photocurrent was focused on the source and drain regions. For the PMOS device, in addition to the source and drain regions, the photocurrent in the N-well region was also considered.

**Figure 5:**
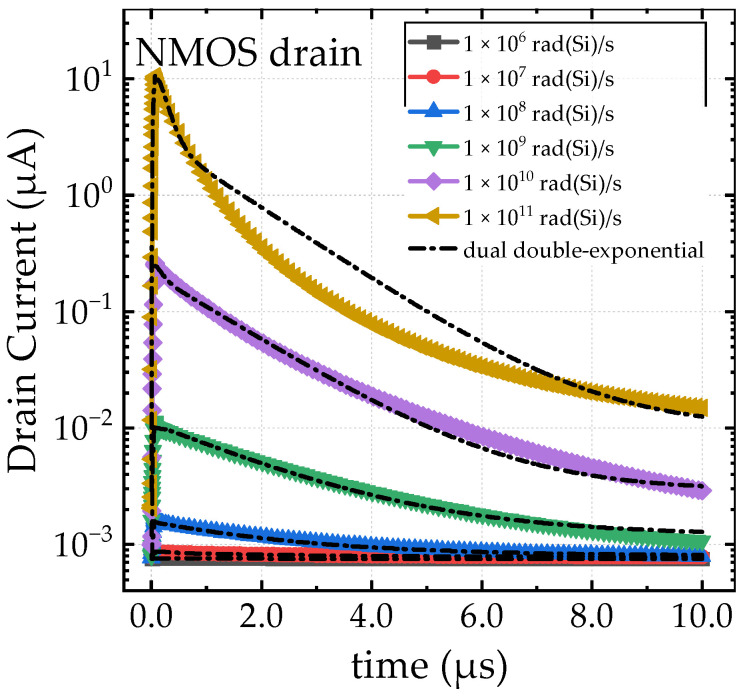
The photocurrent of the NMOS device under different dose rates.

The photocurrent profiles of the NMOS device are shown in Figure 5. Each profile exhibits a pronounced peak current, denoted as *I*_peak_, which increases with the dose rate. For instance, at a dose rate of 1 × 10^11^ rad(Si)/s, *I*_peak_ reaches 1 × 10^−5^ A, which is four orders of magnitude higher than that observed at 1 × 10^6^ rad(Si)/s. Additionally, the photocurrent profiles display a significant diffused component and exhibit a clear tailing characteristic.

**Figure 6:**
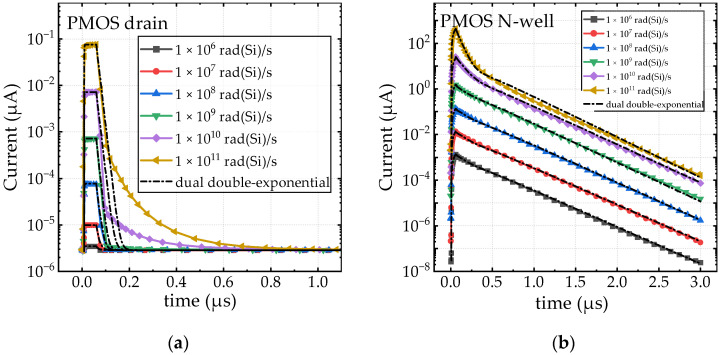
The photocurrent of the PMOS device under different dose rates in the drain (**a**) and N-well (**b**) regions.

The photocurrent profiles of the PMOS device are shown in Figure 6, with Figure 6a representing the drain region and Figure 6b representing the N-well region. The photocurrent profile is heavily dependent on factors such as pulse duration, doping concentrations, and geometric volumes. While the overall trends in the photocurrent results for the NMOS and PMOS devices are consistent, some differences in the details were observed. For example, compared to the NMOS device, the photocurrent in the drain region of the PMOS device is smaller, with a shorter duration. This can be attributed to a reduction in the effective charge collection volume and a shorter diffusion length. Conversely, due to its larger charge collection volume, the photocurrent in the N-well region of the PMOS device exhibits the largest amplitude, approximately three orders of magnitude higher than in the drain region at the same dose rate.

#### 2.2.2. Analysis of the Processing of Photocurrents Induced by the TDRE

The SPICE simulator is capable of handling complex nonlinear circuits and supports a wide range of analysis functions, including DC analysis, AC analysis, transient analysis, and Monte Carlo analysis, among others. However, there is no built-in radiation model in the SPICE simulator. In order to carry out TDRE simulation at the circuit level, it is necessary to reasonably introduce the transient photocurrents obtained from TCAD simulation into the SPICE simulator. Therefore, the double-exponential function is used to model transient photocurrents, which is a standard current model in the SPICE simulator and has been widely used in previous papers [28,29]. The *I*(*t*) of the double-exponential function is given by Equation (3):

(3) $$I(t)=\left\{\begin{array}{l} 0;\;\;\;\;\;\;\;\;\;\;\;\;\;\;\;\;\;\;\;\;\;\;\;\;\;\;\;\;\;\;\;\;\;\;\;\;\;\;\;\;\;\;\;\;\;\;\;\;\;\;\;\;\;\;t<t_{d1}\\ I_{peak}(1-e^{-\frac{\left(t-t_{d1}\right)}{\tau_{1}}});\;\;\;\;\;\;\;\;\;\;t_{d1}<t<t_{d2}\\ I_{peak}(e^{-\frac{\left(t-t_{d2}\right)}{\tau_{2}}}-e^{-\frac{\left(t-t_{d1}\right)}{\tau_{1}}});\;\;\;\;t>t_{d2} \end{array}\right.$$

In this context, *t_d_*_1_ and *t_d_*_2_ represent the start times of the rise and fall of the photocurrent pulse, respectively; *τ*_1_ and *τ*_2_ are the time constants for the rise and fall of the photocurrent pulse; and *t* denotes time.

The fitting results for the photocurrent waveforms in Figure 5 and Figure 6 are provided in Table 1 and Table 2. For different photocurrent waveforms, a single double-exponential function or a combination of two double-exponential functions may be required for fitting, depending on the characteristics of the waveform [28,29]. Specifically, the drain photocurrents of the PMOS are fitted using a single double-exponential function, with the data presented in Table 1. In contrast, the N-well photocurrents of the PMOS and the drain photocurrents of the NMOS are fitted using two double-exponential functions: one short double-exponential component for the prompt part of the photocurrent and one long double-exponential component for the long tail. The fitting data for these cases are shown in Table 2.

From the fitting results, it can be observed that some parameters remain unchanged with varying dose rates. Notably, for the PMOS drain photocurrent fitting data, only the peak fitting parameter *I_peak_* increases with dose rate, while the time-related parameters remain constant. For the NMOS drain and PMOS N-well fittings, the parameter variations are more complex due to the superposition of the two double-exponential functions. It is important to note that the double-exponential functions are simply mathematical tools used to fit and characterize the photocurrent waveforms, facilitating circuit-level simulations. The parameters obtained from the fitting process do not have direct physical interpretations.

## 3. Results and Discussion

**Figure 7:**
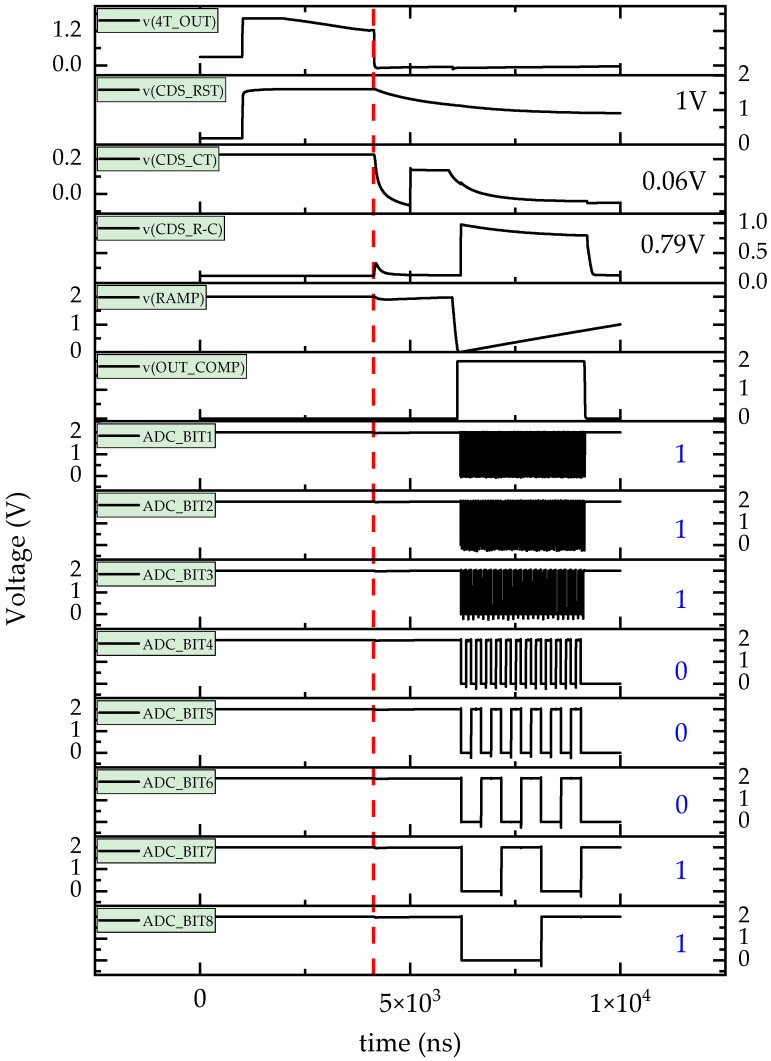
Simulation results of typical CMOS sensor readout circuit output under 1 × 10^10^ rad(Si)/s.

Using the double-exponential current source fault injection method, the TDRE on the readout circuit of CISs (CMOS image sensors) was studied. As shown in Figure 7, typical conditions for the TDRE on the CIS readout circuit were tested, with a dose rate of 1 × 10^10^ rad(Si)/s and a fault injection moment at 4130 ns. The red dotted line in Figure 7 marks the point at which the TDRE occurs. Compared to the non-irradiated state, the TDRE impacted several signals, including V_4T_OUT_, V_CDS_RST_, V_CDS_CT_, V_CDS_R−C_, V_OUT_COMP_, and V_4T_OUT_ADC_. For example, the V_4T_OUT_ signal rapidly decreased at 4130 ns, causing the V_CDS_RST_ signal to drop from 1.605 V to approximately 1V and the V_CDS_CT_ signal to fall from 1.223 V to 0.06 V. Additionally, the sharp decrease in V_4T_OUT_ caused the V_CDS_R−C_ signal to increase from 0.39 V (before the TDRE) to 0.79 V (after the TDRE). The V_4T_OUT_ADC_ signal also changed from “01100001” to “11000111”, and the corresponding V_4T_OUT_ADC_10_ value after the TDRE increased to 0.78 V, nearly doubling from 0.38 V before irradiation. The simulation results showed that when the dose rate was increased to 1 × 10^10^ rad(Si)/s, the TDRE enhanced the output signal of the CIS readout circuit. This is reflected in an increase in the brightness of the pixel unit, as seen in the imaging characteristics.

**Figure 8:**
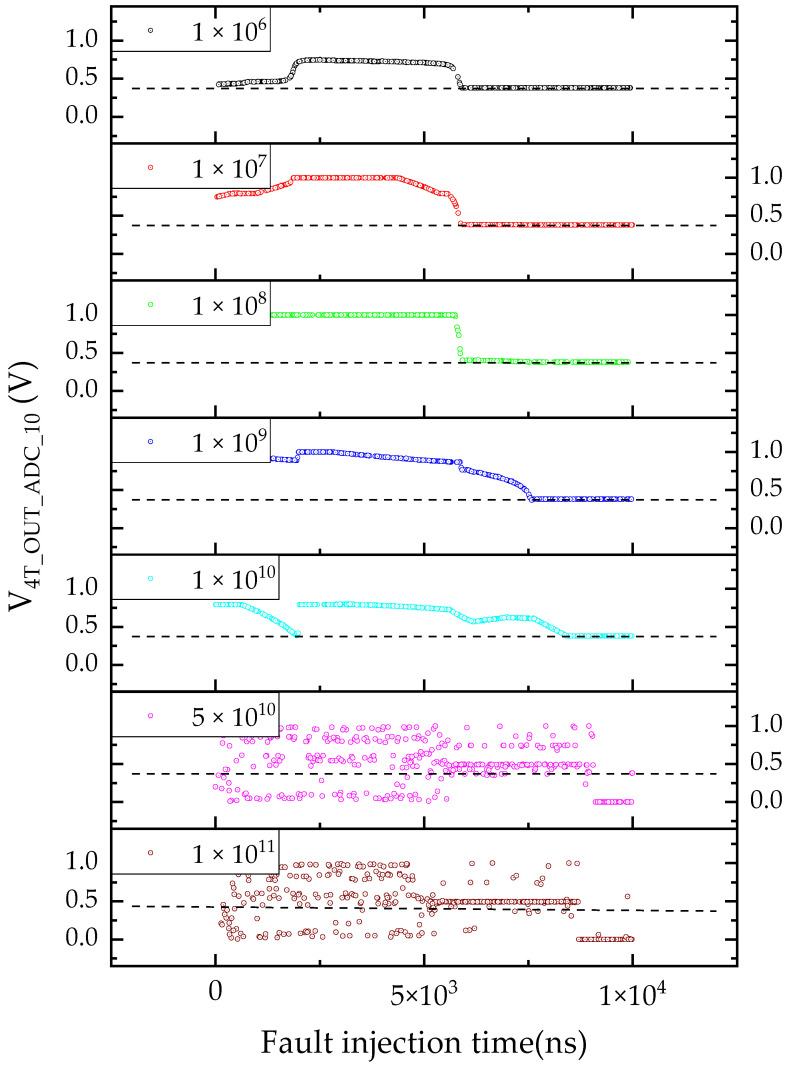
The value of *V*_4T_OUT_ADC_10_ under different fault injection times and dose rates.

In addition, the value of V_CDS_R−C_ (0.79 V) obtained from the subtractor circuit was nearly equal to the value of V_4T_OUT_ADC_10_ (0.78 V), indicating that under these conditions, the TDRE had minimal influence on the ADC circuit. Furthermore, the impact of the moment when the TDRE occurred on the CIS readout circuit was investigated. As shown in Figure 8, simulation results for different dose rates (1 × 10^6^, 1 × 10^7^, 1 × 10^8^, 1 × 10^9^, 1 × 10^10^, 5 × 10^10^, and 1 × 10^11^ rad(Si)/s) are presented. It should be noted that, for easier data analysis and processing, the results in Figure 8 focus on the V_4T_OUT_ADC_10_ signal. The black dotted line represents the output value of the CIS readout circuit in the non-irradiated state, which is approximately 0.39 V.

The results showed that, under low dose rates (≤1 × 10^10^ rad(Si)/s), the CIS readout circuit could successfully acquire and digitally process photosensitive signals. In this range, the value of V_4T_OUT_ADC_10_ was greater than or equal to the non-irradiated value. Preliminary analysis indicated that the change in the value of V_4T_OUT_ADC_10_ was primarily related to the 4T pixel unit and CDS. When the fault injection time was less than 6 × 10^3^ ns, the CIS readout circuit’s function focused on the 4T pixel unit exposure and the double sampling of V_4T_OUT_. During this phase, the introduction of *I*(*t*) into the CIS readout circuit’s compact model accelerated the discharge rate of the integral capacitance of both the 4T pixel unit and CDS, which, in turn, caused the value of V_CDS_R−C_ to increase compared to the non-irradiated condition, as shown in Figure 7.

When the fault injection time exceeded 6 × 10^3^ ns, the CIS readout circuit’s function shifted to converting the V_CDS_R−C_ signal to a machine-readable V_4T_OUT_ADC_ signal. As previously mentioned in Figure 7 with a dose rate of 1 × 10^10^ rad(Si)/s, the TDRE had a minimal impact on the ADC circuit, and the value of V_4T_OUT_ADC_10_ was approximately equal to the non-irradiated value. In contrast, for dose rates greater than 1 × 10^10^ rad(Si)/s, the value of V_4T_OUT_ADC_10_ became randomly distributed between 0 and 1 V, exhibiting considerable impact. In terms of imaging characteristics of considerable impact, several random phenomena occurred in the pixel units, such as loss of photoelectric signal information, luminance darkening, lighting instability, and failure to change, which aligned with experimental observations of transient bright spots or lines [30,31]. These phenomena were primarily attributed to the ADC circuit’s response. The introduction of I(t) into the CIS readout circuit’s compact model could lead to unexpected reversal or interruption of the ADC counter circuit, resulting in a reduction in the specific signal value or complete failure, with no effective signal output.

**Figure 9:**
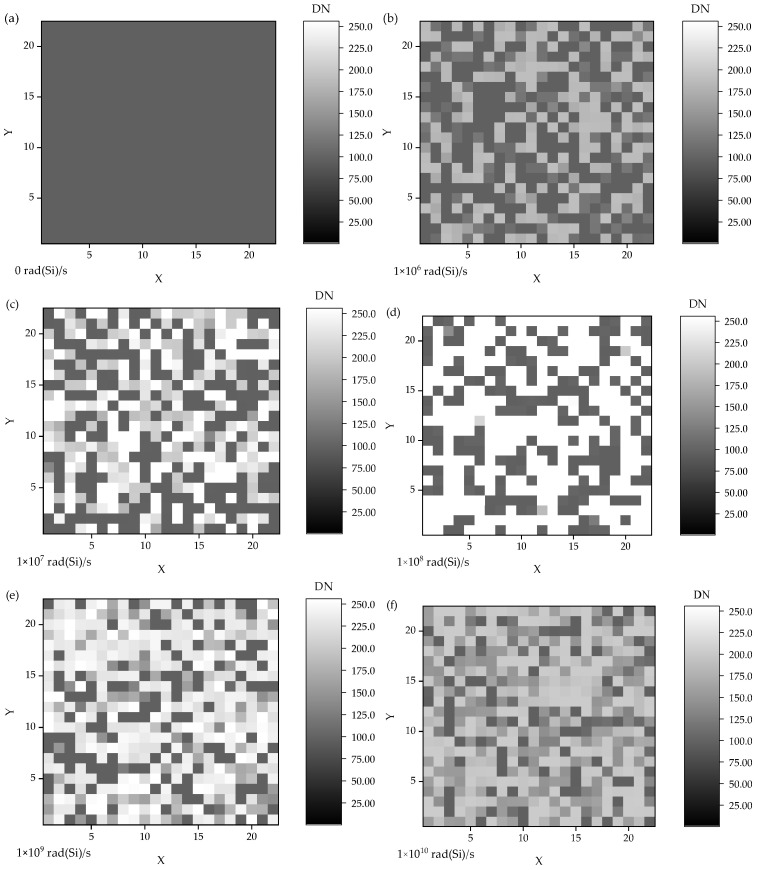

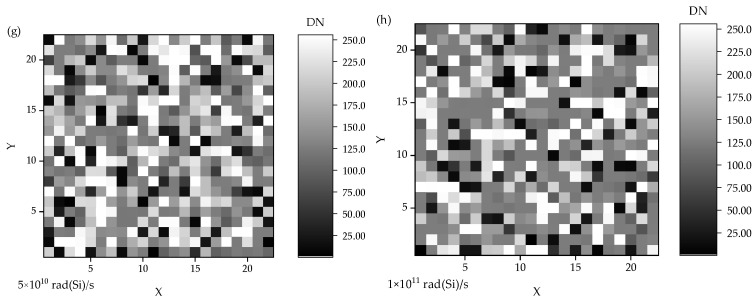
The influence of the TDRE on the CIS image readout circuit from the aspect of imaging characteristics, under different dose rates: (**a**) 0, (**b**) 1 × 10^6^ rad(Si)/s, (**c**) 1 × 10^7^ rad(Si)/s, (**d**) 1 × 10^8^ rad(Si)/s, (**e**) 1 × 10^9^ rad(Si)/s, (**f**) 1 × 10^10^ rad(Si)/s, (**g**) 5 × 10^10^ rad(Si)/s, and (**h**) 1 × 10^11^ rad(Si)/s.

Based on the simulation results of different occurrence time and dose rate, the influence of TDRE on CISs readout circuit was studied in the aspect of imaging characteristics. As shown in Figure 9a–h, after conducting nearly to be 500 times simulations, all possible existence of pixel unit luminance value under different dose rates, such as 0, 1 × 10^6^, 1 × 10^7^, 1 × 10^8^, 1 × 10^9^, 1 × 10^10^, 5 × 10^10^, and 1 × 10^11^ rad(Si)/s, was respectively given out using a constructed 23 × 23 array. In the Figure 9a, the corresponding signal level was 97 DN, which was under in non-irradiated conditions.

The results showed that, under low dose rates, the pixel unit luminance value increased overall, which was consistent with experimental observations [31]. In contrast, under high dose rates, the pixel unit luminance exhibited significant variability due to unexpected flipping or interruption of the ADC circuit, leading to transient bright spots or lines in the image. Therefore, the timing of the TDRE was a crucial influencing factor on the CIS readout circuit and could not be ignored.

Additionally, in extreme γ radiation environments, CISs may experience more severe transient gamma rays, potentially causing permanent damage or even burnout. Furthermore, strong electromagnetic interference often accompanies radiation environments, which can also impact the stable operation of CISs. Further research is needed to fully understand these effects and their implications.

## 4. Conclusions

The dose rates and occurrence timing of the TDRE on the CIS readout circuit were carefully considered in this study. The results demonstrated that, at low dose rates, the readout circuit was able to perform data acquisition and digital processing, with the value of V_4T_OUT_ADC_10_ showing a degree of sensitivity to the occurrence timing. This sensitivity was primarily related to the working principle of the 4T pixel unit and the CDS (correlated double sampling) process. At high dose rates, however, the value of V_4T_OUT_ADC_10_ not only showed sensitivity to the occurrence timing but also exhibited significant variability. This variability was mainly attributed to unexpected flipping or interruptions in the ADC circuit. The timing of the TDRE occurrence thus proved to be a critical factor influencing the performance of the CIS readout circuit and could not be overlooked. Based on the simulation results of the CIS readout circuit, this study provides valuable insights and references for further research on the TDRE in such circuits. Future studies on the dose rate effect of CISs are necessary, and exploring the feasibility of laser irradiation as a method for studying the dose rate effect could provide a viable approach to overcoming challenges in conducting CIS dose rate experiments.

## Figures and Tables

**Table 1:** Fitting parameters of double-exponential functions in Figure 6a.

Dose Rate (rad(Si)/s)	*I_peak_* (A)	*τ*_1_ (ns)	*τ*_2_ (ns)	*t_d_*_1_ (ns)	*t_d_*_2_ (ns)
1.0 × 10 ^6^	3.5 × 10^−12^	1	10	8	60
1.0 × 10 ^7^	9.9 × 10^−12^	1	10	8	60
1.0 × 10 ^8^	7.7 × 10^−11^	1	10	8	60
1.0 × 10 ^9^	7.2 × 10^−10^	1	10	8	60
1.0 × 10 ^10^	7.7 × 10^−9^	1	10	8	60
1.0 × 10 ^11^	7.6 × 10^−8^	1	10	8	60

**Table 2:** Fitting parameters of dual double-exponential functions in Figure 5 and Figure 6b (note: “s” refers to the short component of the dual double-exp, and “l” refers to the long component of the dual double-exp).

Dose Rate (rad(Si)/s)	NMOS/PMOS		*I_peak_*_s (A)	*I_peak_*_l (A)	*τ*_1__s (ns)	*τ*_2__s (ns)	*τ*_1__l (ns)	*τ*_2__l (ns)
1.0 × 10^6^	NMOS	Drain	1.5 × 10^−11^	7.7 × 10^−12^	60	90	120	2500
	PMOS	N-well	2.1 × 10^−9^	1.1 × 10^−9^	50	80	120	270
1.0 × 10^7^	NMOS	Drain	1.5 × 10^−10^	7.6 × 10^−11^	60	90	61	2500
	PMOS	N-well	2.1 × 10^−8^	1.05 × 10^−8^	50	80	120	270
1.0 × 10^8^	NMOS	Drain	2.3 × 10^−9^	7.5 × 10^−10^	60	60	61	2400
	PMOS	N-well	2.1 × 10^−7^	1.1 × 10^−7^	50	80	120	265
1.0 × 10^9^	NMOS	Drain	2.0 × 10^−8^	9.5 × 10^−9^	70	60	57	2100
	PMOS	N-well	2.4 × 10^−6^	1.1 × 10^−6^	50	70	100	255
1.0 × 10^10^	NMOS	Drain	5.0 × 10^−7^	2.0 × 10^−7^	70	90	95	1500
	PMOS	N-well	3.9 × 10^−5^	6.0 × 10^−6^	50	70	120	260
1.0 × 10^11^	NMOS	Drain	1.9 × 10^−5^	3.0 × 10^−6^	70	150	100	1400
	PMOS	N-well	6.7 × 10^−4^	2.0 × 10^−5^	50	50	120	245

## Data Availability

Data are contained within the article.
